# Histiocytose langerhansienne pulmonaire révélée par un pneumothorax: à propos d’un cas

**DOI:** 10.11604/pamj.2016.25.32.10356

**Published:** 2016-09-27

**Authors:** Hafsa Sajiai, Mariam Rachidi, Hind Serhane, Salma Aitbatahar, Lamyae Amro

**Affiliations:** 1 Service de Pneumologie, Hôpital Arrazi, CHU Mohamed VI, Laboratoire PCIM, UCAM, Marrakech, Maroc

**Keywords:** Histiocytose, pneumothorax, kyste, Histiocytosis, pneumothorax, cyst

## Abstract

L’histiocytose langerhansienne est une affection rare d’étiologie inconnue caractérisée par une infiltration d’un ou plusieurs organes, par des cellules de type Langerhans. Elle a une présentation clinique polymorphe. Nous rapportons le cas de Mr R.Y, âgé de 22 ans, tabagique à 8 PA, admis pour pneumothorax total spontané droit. Un drainage thoracique a été réalisé avec bonne évolution. La TDM thoracique de contrôle a objectivé de multiples formations kystiques diffuses prédominant aux lobes supérieurs. Un bilan a été réalisé à la recherche d’une histiocytose systémique mais s’est révélé négatif. L’évolution était marquée par la récidive du pneumothorax, le recours à une pleurodèse et la réalisation d’une biopsie pulmonaire qui a confirmé le diagnostic. Le diagnostic de l’HistiocytoseLangerhansienne doit être évoqué devant un pneumothorax sur poumon kystique. Le diagnostic est aisé devant un tableau clinique et radiologique évocateur. Néanmoins, les possibilités thérapeutiques restent limitées et la récidive du pneumothorax est fréquente.

## Abstract (en)

Langerhans cell histiocytosis is a rare disease of unknown etiology characterized by the infiltration of Langerhans cells in one or more organs. It has a polymorphic clinical presentation. We report the case of Mr R.Y, age 22, with 8 pack year history of smoking, admitted to hospital with complete spontaneous right-sided pneumothorax. Chest drainage was performed with good evolution. Control chest CT scan showed multiple diffuse cyst formations, predominant in the upper lobes. Lab and imaging tests were performed in order to detect systemic histiocytosis with negative results. Patient’s evolution was marked by pneumothorax recurrence; pleurodesis and lung biopsy were performed which confirmed the diagnosis. The diagnosis of Langerhans cell histiocytosis should be evoked in front of pneumothorax associated with lung cystic. The diagnosis is easy in front of a suggestive clinical and radiological picture. Nevertheless, therapeutic options are limited and pneumothorax recurrence is common.

## Introduction

L’Histiocytose Langerhansienne (HL), anciennement appelée Histiocytose X, est une affection rare, d’étiologie inconnue, qui touche les sujets jeunes des deux sexes, avec une prédominance masculine. Elle est caractérisée par l’infiltration des tissus par des cellules dendritiques qui présentent les caractéristiques phénotypiques des cellules de Langerhans, le plus souvent organisées en granulome. Tous les organes peuvent être atteints, mais les lésions touchent essentiellement l’os, le poumon, la peau et l’axe hypothalamo hypophysaire. Histiocyte Society classe les formes cliniques de l’HL selon le nombre et le type d’organes atteints [1]. Le pronostic dépend essentiellement de l’étendue des lésions et des conséquences fonctionnelles. L’atteinte pulmonaire peut être présente au cours d’une forme systémique d’HL. Elle survient souvent chez l’adulte jeune, sous forme isolée. Elle fait partie des pneumopathies infiltrantes diffuses kystiques.

## Patient et observation

Mr R.Y, âgé de 22 ans, porteur de charges de profession, tabagique à 8PA, consommateur de Cannabis, ayant comme antécédents une toux productive, une dyspnée stade III de Sadoul et des infections respiratoires à répétition à raison de 8 épisodes/an depuis 2 ans. Aucun cas de pneumothorax familial n’a été retrouvé.

Le patient avait présenté de façon brutale une douleur basithoracique droite spontanée, en coup de poignard, associée à une dyspnée stade V de Sadoul et à une toux productive ramenant des expectorations blanchâtres, le tout évoluant dans un contexte d’apyrexie et de conservation de l’état général. L’examen clinique a trouvé un tirage sus sternal, un DEP à 170 L/min, un syndrome d’épanchement aérique de tout l’hémithorax droit et un hippocratisme digital et des orteils manifeste.

**Figure 1:**
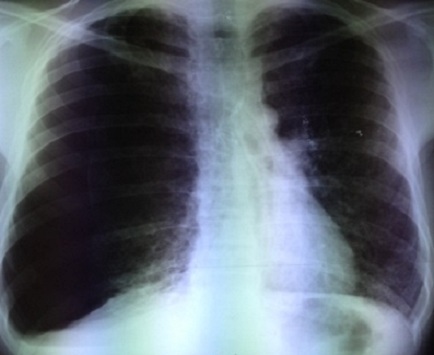
Radiographie thoracique montrant un pneumothorax total droit sur poumon kystique

**Figure 2:**
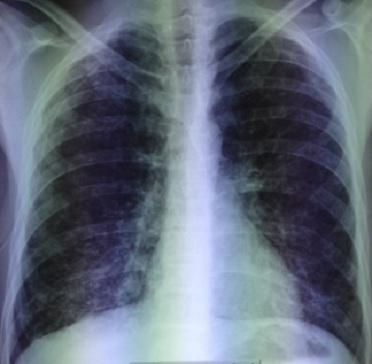
Radiographie thoracique après drainage montrant des lésions kystiques bilatérales

La radiographie du thorax a montré une hyper clarté sans trame vasculaire de l’hémithorax droit en faveur d’un pneumothorax de grande abondance. Le parenchyme pulmonaire gauche est siège d’images kystiques, de taille variable, diffuses, qui prédominent aux territoires supérieurs avec une distension thoracique (Figure 1). Un drainage thoracique a été réalisé au niveau du 4ème espace intercostal droit. La radiographie thoracique de contrôle a montré un retour du poumon à la paroi. Les lésions kystiques étaient plus visibles après drainage et étaient bilatérales (Figure 2).

**Figure 3:**
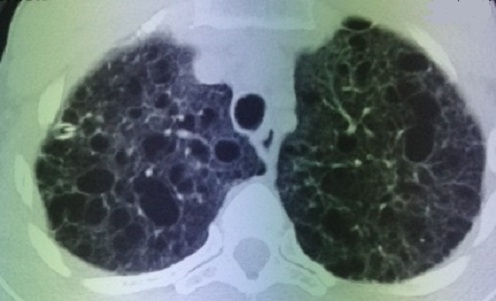
TDM thoracique montrant des lésions pulmonaires kystiques bilatérales

Un complément scannographique a été demandé afin de mieux explorer les images kystiques. Il s’agissait de multiples formations kystiques diffuses prédominant au niveau des lobes supérieurs, de taille variable, à parois fines et confluentes par endroits. Il s’y associait un épaississement des septas inter lobulaires et sous pleural diffus et un aspect en verre dépoli au niveau des deux pyramides basales. Des lésions micronodulaires à contours flous intra parenchymateux et en sous pleural à contours flou étaient également retrouvées, intéressant le segment ventral du lobe supérieur, le lingula, le segment latéral du lobe moyen et les deux pyramides basales. L’aspect scannographique était en faveur d’une HistiocytoseLangerhansienne (Figure 3). Une bronchoscopie a été réalisée avec lavage broncho alvéolaire LBA et biopsies étagées à droite et à gauche. L’aspect endoscopique était normal ainsi que les résultats des biopsies faites et du LBA.

Un bilan de retentissement a été fait: la pléthysmographie a montré un trouble ventilatoire obstructif sévère non réversible après bronchodilatateurs avec une Tiffeneau <0,7, un VEMS après BD de 1,85L soit 45% de la théorique et une importante distension thoracique (VR/ CPT 216%); la gazométrie était normale; le TM6M a mis en évidence une distance parcourue correspondant à 70% de la théorique.

Un bilan à la recherche des autres manifestations systémiques de l’Histiocytose a été fait: radiographies du thorax, bassin, crâne et panoramique dentaire: aucune lyse osseuse n’a été retrouvée; scintigraphie osseuse: normale; l’examen dermatologique et des aires ganglionnaires était normal; l’échographie abdominale a montré une hépatomégalie homogène sans nodules palpables.

Un bilan biologique a été fait et a montré un syndrome inflammatoire biologique sans hyperleucocytose ni anomalies des autres lignées.

L’évolution a été marquée par une récidive homolatérale du pneumothorax. Une pleurodèse droite ainsi qu’une biopsie pulmonaire chirurgicale ont été réalisés. La biopsie a montré un granulome à cellules de Langerhans confirmant ainsi le diagnostic de l’Histiocytose Langerhansienne pulmonaire.

La prise en charge a consisté en un sevrage tabagique. Une corticothérapie inhalée associée à un Beta 2 mimétique de longue durée d’action ainsi qu’une vaccination antigrippale et antipneumococcique.

## Discussion

L’histiocytose langerhansienne recouvre une série d’entités de présentation clinique et de pronostic très variés qui ont en commun sur le plan histologique une infiltration importante des tissus concernés par des cellules de Langerhans, le plus souvent organisées en granulome. Chez l’adulte, les formes localisées de l’histiocytose langerhansienne sont dominées par l’atteinte osseuse et pulmonaire. Les formes pluritissulaires ont une présentation clinique très variable selon les organes atteints : principalement l’os, le poumon, la peau, l’axe hypothalamo-hypophysaire, plus rarement le système hématopoïétique, le foie ou le système nerveuxcentral.

L’HL pulmonaire isolée survient essentiellement chez l’adulte jeune avec un pic de fréquence entre 20 et 40 ans, ce qui est le cas de notre patient. Plus de 90% des patients adultes atteints d’histiocytose langerhansienne pulmonaire fument. S’il existe des arguments pour un rôle déclenchant du tabac, ses effets sur le devenir de la maladie sont moins bien compris [2].

La présentation clinique de l’HL pulmonaire de l’adulte est polymorphe. Elle peut être asymptomatique et découverte à l’occasion d’une radiographie thoracique systématique dans 25% des cas. Elle peut être découverte, comme dans notre observation, à l’occasion d’un pneumothorax spontané dans environ 20% des cas. L’antécédent de toux chronique, de dyspnée stade III et d’infections respiratoires à répétition concordent avec le tableau. L’examen clinique est généralement normal. L’hippocratisme digital est exceptionnel [3].

Sur le plan radiologique, le cliché standard est le plus souvent anormal (90%) et montre des anomalies réticulo-nodulaires qui prédominent dans les parties moyennes et supérieures des poumons. Au scanner thoracique, les lésions élémentaires varient avec l’ancienneté de la maladie.

L’anomalie la plus fréquemment retrouvée en pléthysmographie est un piégeage aérique dans 45% des cas, suivi d’un trouble ventilatoire obstructif dans 30% des cas puis d’un restrictif dans 6% des cas. Une baisse de la DLCO serait notée chez 80% des patients [**3**]. Ceci concorde avec les résultats de la pléthysmographie de notre patient où une augmentation du volume résiduel (VR) et un rapport VR/CPT à 234% et 227% respectivement étaient observés.

L’hématose au repos est longtemps conservée chez ces patients, ce qui en témoigne les résultats gazométriques normaux de notre patient contrastant avec les lésions kystiques évoluées à la TDM thoracique. Le test de marche de 6 min est également normal reflétant la bonne tolérance à l’effort surtout en absence d’HTAP.

La part relative des formes pluritissulaires d’HL est mal connue chez l’adulte. Elle peut être associée à des atteintes extrathoraciques essentiellement osseuses, cutanée ou hypothalamo-hypophysaires [2].

L’atteinte osseuse représente environ 50% des localisations de l’HL de l’adulte. L’aspect radiologique évocateur est celui d’une lyse osseuse à l’emporte-pièce sans condensation périphérique. Il n’a pas été démontré l’intérêt d’un bilan radiologique exhaustif en absence de signes d’appel extra pulmonaires et la place de la scintigraphie osseuse au Tc 99m reste très discutée [4].

L’atteinte cutanée est polymorphe dans l’HL. Les lésions sont souvent papulaires ou vésiculaires, souvent purpuriques siégeant essentiellement dans le tronc, face, cuir chevelu et les plis. Une atteinte gingivale inflammatoire ou nécrotique peut également s’observer. Elle est habituellement associée à une atteinte osseuse maxillaire sous-jacente. L’examen dermatologique minutieux n’a pas objectivé de lésions en faveur d’une HL.

L’atteinte endocrine est dominée par l’atteinte hypothalamo-hypophysaire. Le diabète insipide est la manifestation endocrinienne la plus fréquemment retrouvée [5].

Une fois le diagnostic d’HL posé, un bilan médical limité mais précis doit être réalisé quel que soit le mode de découverte de la maladie. Le but de ce bilan est de déterminer l’extension de l’HL, afin d’adapter le traitement et le rythme de suivi. Ce bilan comporte : un examen clinique complet, hémogramme, bilan hépatique, rénal, ionogramme sanguin, CRP, TSH, T4 libre et dosage de l’osmolarité sanguine et urinaire. Le bilan radiologique comporte des radiographies du squelette entier avec un panoramique dentaire, et une échographie abdominale. La place de la scintigraphie osseuse au Technétium 99 reste discutée, elle garde une place limitée dans cette indication [6]. La TEP-TDM n’est pas systématique, elle semble utile pour évaluer l’activité des lésions osseuses et la réponse précoce au traitement mais elle a été peu évaluée [7]. L’échographie cardiaque garde une grande place dans la recherche d’une HTAP. D’autres examens peuvent être réalisés fonction des signes appels.

La confirmation du diagnostic est histologique par biopsie d’un tissu atteint. La lésion histologique évocatrice est un granulome à cellules de Langerhans. Les cellules de Langerhans ont un noyau contourné, un cytoplasme pâle, faiblement éosinophile et sont donc facilement reconnues en microscopie optique. Leur nature langerhansienne est confirmée par immunohistochimie grâce à leur expression de l’antigène membranaire CD1a ou de la Langerine (CD207). L’aspect du granulome varie en fonction du stade évolutif et du tissu concerné [3].

Le pronostic de la maladie dépend du nombre d’organes atteints et de la sévérité des lésions. L’évolution de l’HL pulmonaire est très variable, globalement imprévisible avec parfois une évolution par poussées. A 5 ans, la fonction respiratoire s’altère chez environ 50% des patients. Parfois, la maladie peut régresser mais la fonction ventilatoire continue de s’aggraver du fait d’une BPCO post-tabagique. Le pneumothorax a tendance à récidiver. Une HTAP peut survenir au cours du suivi d’une HL, en moyenne 10 ans après le diagnostic, ou être découverte de façon concomitante et constitue un facteur pronostique péjoratif. Le pronostic est réservé chez notre patient vue la découverte de la maladie d’emblée à sa phase kystique et la profonde altération de la fonction respiratoire avec un VEMS à 45% de la théorique.

La vinblastine est le traitement de référence des formes systémiques d’HL. Bien que ce traitement soit utilisé chez l’adulte depuis de nombreuses années, très peu d’études permettent de quantifier son efficacité et sa tolérance. Devant les effets secondaires, principalement neurologiques, certains auteurs privilégient l’utilisation d’autres chimiothérapies telle que la cladribine ou la cytarabine [8].

Dans l’HL pulmonaire, aucun traitement n’a fait preuve de son efficacité. La première mesure est d’obtenir un sevrage tabagique qui limite les risques de la BPCO, des pathologies cardiovasculaires et de survenue de cancer bronchique. L’abstention thérapeutique est la règle chez les patients peu ou pas symptomatiques. L’association de corticoïdes et de B2-mimétiques de longue durée d’action par voie inhalée peu améliorer la fonction ventilatoire notamment en cas d’hyperréactivité bronchique. La corticothérapie orale est efficace sur les signes généraux. Elle est prescrite de façon empirique dans les formes pulmonaires récentes, nodulaires, symptomatiques de l’adulte, dans le but d’accélérer la résolution des lésions inflammatoires, mais sans preuve d’efficacité. De rares observations récentes ont suggéré l’efficacité, parfois spectaculaire, de la cladribine (2-CDA) cytostatique, analogue des purines, qui peut être active même dans les formes kystiques diffuses d’HL pulmonaire de l’adulte permettant une amélioration spectaculaire de la fonction ventilatoire chez un petit nombre de patients [9].

Le pneumothorax relève d’un traitement spécifique (drainage et souvent accolement pleural), en évitant si possible la pleurectomie chez ces patients jeunes qui peuvent éventuellement bénéficier d’une transplantation pulmonaire [10]. La vaccination anti-pneumococcique et antigrippale sont souhaitables pour éviter la décompensation respiratoire. La réhabilitation respiratoire permet d’améliorer la qualité de vie de ses patients. Les patients au stade d’insuffisance respiratoire et souvent une HTAP relèvent d’une prise en charge usuelle (oxygénothérapie, diurétiques). La prostacycline par voie veineuse doit être évitée chez ces patients à cause du risque d’œdème pulmonaire, du fait de l’atteinte veinulaire fréquente de leur HTAP.

## Conclusion

Nous rapportons dans cette observation un cas d’histiocytose langerhansienne pulmonaire à sa phase kystique révélée par un pneumothorax chez un jeune patient de 22 ans et confirmée par biopsie pulmonaire. Le patient est peu symptomatique sur le plan clinique malgré la richesse des anomalies scannographiques et spirométriques. Les possibilités thérapeutiques par ailleurs restent très limitées, d’où l’impact psychosocial de cette pathologie. La place de la cladribine (2-CDA) dans les formes évolutives reste à prouver.
